# Retrospective study of a serie of pterygoid implants

**DOI:** 10.4317/medoral.26633

**Published:** 2024-06-22

**Authors:** Pablo Cea-Arestín, Arturo Bilbao-Alonso, Marta Hernández-DeOliveira

**Affiliations:** 1 ORCID ID: 0000-0002-0655-0412. Associate. Department of Surgery and Medical-Surgical Specialties, School of Medicine and Dentistry, University of Santiago de Compostela, Santiago de Compostela, Galicia, Spain; 2 ORCID ID: 0000-0002-3224-1007. MD, PhD, PhD, OMS. Maxillofacial Surgery Service, Santiago de Compostela Hospital Complex (CHUS), Galician Health Service (SERGAS). ZAGA Center A Coruña and Lugo, Galicia, Spain; 3 Emeritus professor. Department of Surgery. University Complutense of Madrid, Madrid, Spain

## Abstract

**Background:**

This article aspires to show that pterygoid implants are a magnificent and viable alternative to other posterior implants of the maxilla, especially in cases of atrophy.

**Material and Methods:**

This study is based on a retrospective analysis of pterygoid implant data from 2003 to 2023, recollecting the following variables: year of placement, location, shape of the implant, diameter of the implant, length of the implant, torque of the implant, whether or not it was post-extraction, whether or not there was immediate loading, whether or not smoking was present (smoking habit), the brand of the implant and the success/survival or failure/non survival of the implant.

**Results:**

The total of 178 pterygoid implants placed in the 113 patients eligible for the study was analysed by subgroups, with percentage of global success of 98.3% (3 failures).

**Conclusions:**

Pterygoid implants offer biomechanical and success/survival characteristics similar or superior to the so-called conventional implants and avoid a series of surgical and prosthodontic procedures more difficult than the ones required by other implants in many occasions.

** Key words:**Pterygoid, implants, maxilla, atrophy.

## Introduction

Although the rehabilitation of oral architecture and function with implants is a predicTable process and with a high percentage of long-term success, sometimes we have to resort to more heterodox sites to position them. This is the case of atrophic maxilla, for which an implantation in the pterygoid process is proposed. This is a challenge due to the anatomy of the area, the risk factors, the biomechanical implications and the few studies that exist in this regard (1,2), reasons why we put this research as necessary.

Pterygoid implants are those placed through the maxillary tuberosity, the pyramidal process of the palatine bone and the pterygoid process of the sphenoid bone (3-4). Thus, in the glossary of Oral and Maxillofacial Implants (GOMI) this kind of implants are defined as "implant placed through the maxillary tuberosity and into the pterygoid plate" (1).

Anatomically, the pterygoid process is determined by measures of 15 mm, although the angulation of the tuberopyramid-pterygoid column varies depending on whether it is a dentulous or edentulous maxilla. In dentulous maxilla, the angulation is 76,5° mesio-distal and 17,2° buco-palatal respect to the Frankfurt plane; while in edentulous maxilla, the angulation is 67,3° mesio-distal and 14,1° buco-palatal respect to the Frankfurt plane (5). Likewise, it’s spatial position and it’s relationship with pterygoid venous plexus, major palatine artery, internal maxillary artery and anterior palatine nerve should be taken into account (6).

For the positioning of a pterygoid implant, the Tulasne surgical technique is used, consisting in a midcrestal incision in the pterygomaxillary region with a vertical releasing incision mesial to homolateral canine with a full-thickness mucoperiosteal buccal flap (7). The inclination of the implant in relation to the pterygomaxillary area will be 30-55° with apico-palatine direction (8-9), although other studies speak of it’s more verticalized implementation (10).

The indications of this type of implants would be: to avoid grafts in atrophic maxillas (1) and thus be able to carry out rehabilitation in a shorter time (11-14), to avoid cantilever prostheses and provide biomechanical stability (1,14-18), as an aid to short implants and zygomatic implants, and also as a rescue technique (2).

On the other hand, it’s contraindications would be the lack of bone in the pterygomaxillary area plus the same as in the conventional implants. However, they present other difficulties: surgical due to the anatomy of the area, reduced space, inclination/angulation and prosthodontic. Possible complications should also be considered, such as: bleeding from the palatine artery, pterygoid venous plexus haemorrhage, absence of primary stability/migration, trismus, infection and paresthesia. However, only minor complications have been reported therefore is a safe insertion zone (1,18-20).

Continuing with their delimitation, they offer the advantages of: reduced treatment time (14,16,21), no need for donor area, no use of biomaterials, good primary stability and better resistance to forces than conventional implants (17), possibility of immediate loading (14,16,22-23), high predictability (21), lower morbidity (22-24), lower economic cost of treatment (24), and the degree of patient satisfaction is very high (in position, functionality, aesthetics, hygiene and speech terms (11-12,14,16-17,21).

## Material and Methods

This study is based on a retrospective analysis of pterygoid implant data. These implants and data have been carried out and acquired in a private clinic in Santiago de Compostela, capital of Galicia, autonomous region of Spain, southern-western Europe, from 2003 to 2023.

Data collected have been compared with those of other studies, being the inclusion criterion that they have to deal with pterygoid implants, excluding the other types of posterior maxillary implants.

Participants were all subjects who had been fitted with pterygoid implants over the years, with all other types of implants placed excluded. Other criteria for the filter were not taken into account. All gave their written consent and were guaranteed the use of their data anonymously for the research in question.

The variables used were: year of placement, location, shape of the implant, diameter of the implant, length of the implant, torque of the implant, whether or not it was post-extraction, whether or not there was immediate loading, whether or not smoking was present (smoking habit), the brand of the implant and the success/survival or failure/non survival of the implant.

The data sources were the medical records of the patients who entered the study and the data collection was effected in the private practice where the positioning of the implants was carried out.

A sample size was obtained from 113 patients with a total of 178 pterygoid implants.

The coding, mechanization and statistical analysis of the data was executed using the computer and mathematical tool of Excel.

All ethical, health, medical-legal, data processing and administrative aspects were complied based on current provisions.

## Results

The total of 178 pterygoid implants placed in the 113 patients eligible for the study was analysed as follows: percentage of global success, percentage of success at 7 years or more, percentage of success at 5 years or more and less than 7 years, percentage of success at 3 or more years and less than 5 years, percentage of success at less than 3 year, diameter, length, shape, torque, if it was post-extraction or not, if it received immediate load or not, smoking habit (yes, no, ex-smoker), location in the maxilla (right, left) and brand of pterygoid implants. And, among those that fail, common factors were sought by examining all the parameters available. Straightaway, results are presented in detail by subgroups in text and in Tables 1, Table 2, Table 3 and Table 4.

## Discussion

The results obtained in our study on a large sample of pterygoid implants suggest their power by providing a high success in relation to their use, being able to report that they have the same or better success rates than other types of implants (17-18,20,25-27).

Comparing our data with those of other papers dealing with pterygoid implants, we see that the success rates are similar between studies (98.3% for our data), with the advantage that in our research we have a large sample (178 pterygoid implants analysed) in relation to most of the studies analysed.

Following with the theme, data from other studies with which the comparison was made are provided, where the success of pterygoid implants, understood as their survival, was in the range of 80% to 99% (1,3,12-16,19-21,28-33). If we stop at two of the most recent, they provide a success/survival rate of 94.87% (20) and 96.45 (21).

Despite the limitations of the study, such as the contribution of a greater number of variables (as, for example, more toxic habits involved) or larger sample size (1), the placement of pterygoid implants is a very good option in certain cases such as the atrophic postero-superior maxilla (1,11,14) due to it’s good primary stability (16), very high success with minimal complications (17) and good acceptance by patients (33). In statements about pterygoid implants extracted from The American College of Prosthodontists “implants in the pterygoid region offer a scientifically validated and predicTable treatment option”.

## Conclusions

This study aims to show that pterygoid implants are a magnificent and viable alternative to other posterior implants of the maxilla, especially in cases of atrophy. Pterygoid implants offer biomechanical and success/survival characteristics similar or superior to the so-called conventional implants and avoid a series of surgical and prosthodontic procedures more difficult than the ones required by other implants in many occasions.

## Figures and Tables

**Table 1:** Implant data by periods.

Period	Pterygoid implants under study	Success	Failure	Percentage of success (%)
Entire period of study 2003-2023	178	175	3	98.3
With 7 or more years of study 2003-2016	81	79	2	98.8
With 5 to 7 years (with 5 years not included) 2016-2018	54	53	1	98.2
With 3 to 5 years (with 3 years not included) 2018-2020	38	38	0	100
With less than 3 years 2020-2023	5	5	0	100

**Table 2:** Most commonly implant features.

Feature	Quantity from the overall	Percentage from the overall (%)
Most commonly used implant diameter: 4 mm	90	50.6
Most commonly used implant length: 16 mm	108	60.7
Most commonly used form of implant: conical	146	82
Implant torque: between more than 40 and 60 N/cm	100	100

**Table 3:** Post-extraction and immediate load data.

Feature	Yes (% from overall)	No (% from overall)
Post-extraction	44 (24.7)	134 (75.3)
Immediate load	23 (12.9)	155 (87.1)

**Table 4:** Implant brand data.

Implant brand	Number from overall	Percentage from overall (%)
Lasak BioniQ	90	50.5
Straumann	40	22.4
BioHorizons	20	11.2
Nobel Biocare	13	7.3
Zimmer Biomet	9	5.0
Astra Tech	4	2.1
Trinon Q	3	1.5
